# Repurposing ferumoxytol: Diagnostic and therapeutic applications of an FDA-approved nanoparticle

**DOI:** 10.7150/thno.67375

**Published:** 2022-01-01

**Authors:** Yue Huang, Jessica C. Hsu, Hyun Koo, David P. Cormode

**Affiliations:** 1 Department of Radiology, Perelman School of Medicine, University of Pennsylvania, Philadelphia, PA, USA; 2 Biofilm Research Labs, Levy Center for Oral Health, School of Dental Medicine, University of Pennsylvania, Philadelphia, PA, USA; 3 Department of Preventive & Restorative Sciences, School of Dental Medicine, University of Pennsylvania, Philadelphia, PA, USA; 4 Department of Bioengineering, School of Engineering and Applied Sciences, University of Pennsylvania, Philadelphia, PA, USA

**Keywords:** ferumoxytol, magnetic resonance imaging (MRI), iron deficiency, nanozyme, drug delivery

## Abstract

Ferumoxytol is an intravenous iron oxide nanoparticle formulation that has been approved by the U.S. Food and Drug Administration (FDA) for treating anemia in patients with chronic kidney disease. In recent years, ferumoxytol has also been demonstrated to have potential for many additional biomedical applications due to its excellent inherent physical properties, such as superparamagnetism, biocatalytic activity, and immunomodulatory behavior. With good safety and clearance profiles, ferumoxytol has been extensively utilized in both preclinical and clinical studies. Here, we first introduce the medical needs and the value of current iron oxide nanoparticle formulations in the market. We then focus on ferumoxytol nanoparticles and their physicochemical, diagnostic, and therapeutic properties. We include examples describing their use in various biomedical applications, including magnetic resonance imaging (MRI), multimodality imaging, iron deficiency treatment, immunotherapy, microbial biofilm treatment and drug delivery. Finally, we provide a brief conclusion and offer our perspectives on the current limitations and emerging applications of ferumoxytol in biomedicine. Overall, this review provides a comprehensive summary of the developments of ferumoxytol as an agent with diagnostic, therapeutic, and theranostic functionalities.

## Introduction

Iron oxide nanoparticles have been increasingly used in a variety of biomedical applications because of their inherent multifunctional properties, such as superparamagnetic behavior and biocatalytic activity. These nanoparticles can possess different crystal structures including hematite (α-Fe_2_O_3_), maghemite (γ -Fe_2_O_3_), and magnetite (Fe_3_O_4_) 1. Synthetic control over the particle's composition, size, morphology, and surface chemistry has specific impacts on its biodistribution, pharmacokinetics, and suitability for various biomedical uses. In particular, iron oxide nanoparticles with extremely small core sizes ranging between 3 nm and 15 nm have broad applicability and high translational potential 2. Nevertheless, iron oxide cores (particularly magnetite) are easily oxidized in air and are insoluble in aqueous solution. Thus, surface coatings that can maintain iron oxide nanoparticle stability in biological media and prevent the loss of magnetism are necessary 3.

Numerous iron oxide nanoparticle formulations have been studied in both preclinical and clinical settings. Some of these formulations have already appeared on the market 4-7. It is estimated that the global market for biomedically applied magnetic particles will reach US $87.7 million by 2025 and will increase by ⁓10% annually 8. This is largely driven by the growing population of patients with renal diseases as well as increasing demands for the diagnosis and treatment for certain diseases. Therefore, the economic prospects for iron oxide nanoparticles are very good. The following iron oxide nanoparticles have been approved by the U.S. Food and Drug Administration (FDA) for clinical use: Feraheme® for iron deficiency; Combidex® (U.S.) and Sinerem® (Europe) as a magnetic resonance imaging (MRI) agent; Nanotherm® (MagForce) for cancer treatment; and Lumirem® as an oral gastrointestinal tract imaging agent.

Among these iron oxide nanoparticles, Feraheme® (ferumoxytol injectable solution) was approved in the U.S. in 2009, Canada in 2011, and Europe in 2012 and has been used for treating iron-deficiency anemia (IDA), which is often found in renally impaired patients 9. Moreover, ferumoxytol holds great promise for many other biomedical applications including MRI, drug delivery, oral biofilm treatment, and anti-cancer and anti-inflammatory therapies. Notably, ferumoxytol is being used as an MRI contrast agent in ongoing clinical trials 4, 10.

### Physicochemical properties of ferumoxytol

Ferumoxytol is produced by AMAG pharmaceuticals, Inc. (Cambridge, MA). It is an ultrasmall iron oxide nanoparticle that contains a superparamagnetic (ferri- or ferromagnetic) iron oxide core with approximately 2000 magnetite (Fe_3_O_4_) formula units and a hydrophilic carboxymethyl-dextran coating (10 kDa in molecular weight) 11. This surface coating is predominantly composed of linear α-(1,6)-linked glucose polysaccharide with hydrogenated end groups and partially carboxymethylated groups at positions C-2, C-3, or C-4 12. This dextran also has a low degree of α-(1,3) type glucose polysaccharide 12. The coating material is about 1.7 nm thick and provides ferumoxytol with a neutrally charged surface. The inorganic cores of ferumoxytol are approximately 7 nm in diameter 13 and the overall size of ferumoxytol is approximately 17-31 nm in diameter. Ferumoxytol maintains its structure under physiological conditions and its dextran-derived coating minimizes the release of labile iron until the complex enters the reticuloendothelial system (RES) macrophages 14. Ferumoxytol is a sterile liquid at a neutral pH, or an isotonic solution of mannitol 15. When compared with other intravenous iron agents, preliminary data shows that ferumoxytol contains less free iron 16. Thus, it can be considered as safe to be administered quickly in relatively high doses 17. After intravenous administration, ferumoxytol has a slow dissolution rate, which is beneficial for treating IDA, especially in patients with renal impairment, due to a low level of free iron circulating in the blood 18.

### Administration routes of ferumoxytol

Initially, ferumoxytol was approved as an intravenous iron formulation for the treatment of IDA. It is usually administered as a rapid bolus at a rate of 30 mg per ml per second. The dose was determined by a prospective comparison between placebo and ferumoxytol, where no difference in potential serious adverse events (SAEs) was observed 19, 20. For MRI, contrast-enhanced images can be obtained at doses between 1 and 7.5 mg per kg depending on the pulse sequence and type of system used 21. Additionally, our group explored topical ferumoxytol application as a nanozyme to kill bacteria and disrupt biofilm for dental caries (tooth decay) prevention. For this application, we administered ferumoxytol at 1 mg per ml topically in the oral cavity followed by 1% hydrogen peroxide exposure in a rodent model of dental caries 13. Recently, we employed a similar topical treatment regimen to target biofilms causing tooth decay in the human mouth 22.

### Multifunctionality of ferumoxytol

**Figure 1:**
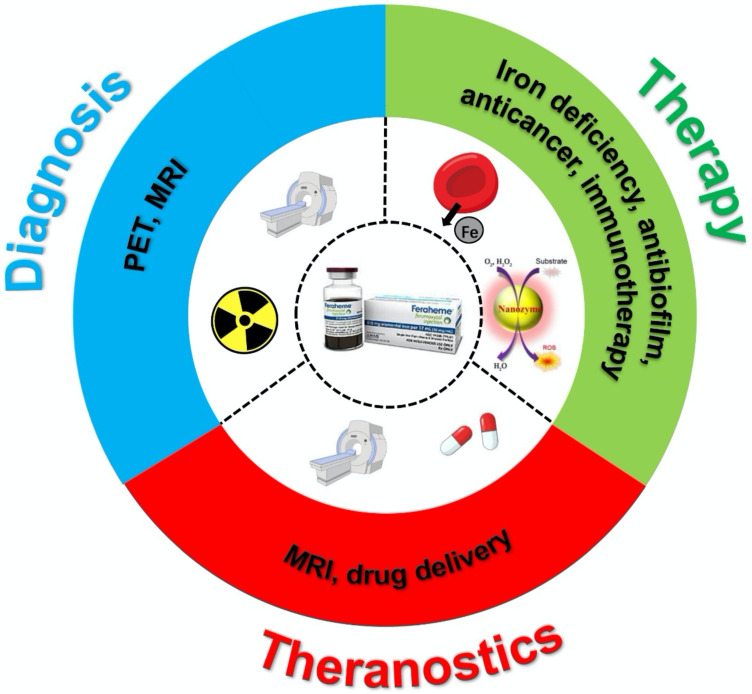
Scheme of ferumoxytol for diagnosis, therapy and theranostics applications.

In general, ferumoxytol has features that are similar to other iron oxide nanoparticles, such as superparamagnetism and biocatalytic activity. First, ferumoxytol can provide therapeutic effects by acting as an iron supplement 23. The efficiency and safety of IDA treatment with ferumoxytol have been reported by numerous studies. Second, ferumoxytol is an ultrasmall superparamagnetic iron oxide nanoparticle that can result in image contrast for MRI via T_1_, T_2_, and T_2_^*^ shortening. Moreover, the carboxymethyl-dextran coating shell allows the nanoparticle to remain in the blood circulation for a long time (half-life ~14.5 hrs) 24. Third, reactive oxygen species (ROS) can be generated when ferumoxytol is present with hydrogen peroxide 25. Therefore, ferumoxytol is a multifunctional iron oxide nanoparticle that can be used in various biomedical applications (Figure 1).

At present, the topic of iron oxide nanoparticles is well discussed in numerous review articles. Most of them focus on synthesis, characterization, and surface functionalization, as well as the biomedical applications and so forth 1, 5, 26, 27. However, to the best of our knowledge, there is no review that highlights the versatility and broad applicability of ferumoxytol thus far. To bridge this gap, we will focus on the recent advances in the diagnostic and therapeutic applications of ferumoxytol in this review. This will prompt us to re-evaluate the role of ferumoxytol in biomedical uses, which, due to its prior FDA approval, would have the potential of being readily adopted in the clinic in the future.

### Ferumoxytol hybrid structures and applications

To expand the applications, ferumoxytol hybrid was also explored based on the inherent structure of ferumoxytol consisting of a superparamagnetic iron oxide core and a shell of carboxymethyl-dextran polymer. In general, these ferumoxytol hybrids were developed through the chemical reaction or physical interaction of carboxymethyl-dextran polymer with other models. It is evidenced that there are several strategies to form the ferumoxytol hybrid based on chemical reactions. One is the use of carboxyl groups on the carboxymethyl-dextran polymer, these carboxyl groups could be further modified and converted to amines, as well as reaction with the NHS esters of other molecules (*e.g.* fluorochromes) 28. This could be used for fluorescence labelling of ferumoxytol for bioimaging. Another approach is using a chelateless radiocation surface adsorption method in which radiocations (*e.g.* ^89^Zr^4+^, ^64^Cu^2+^, etc.) could be doped on ferumoxytol 28. This could be used for radiolabeling of ferumoxytol for dual-modality imaging. In addition, ferumoxytol hybrid could be formed based on physical interaction. The carboxyl groups on the carboxymethyl-dextran carry the negative charge, it could cause physical absorption with other models carrying positive charges through weak electrostatic interaction. This could make small cargos retain within carboxymethyl-dextran coating polymer and could be used for MRI-guided drug delivery 29. On the other hand, ferumoxytol could also cap onto the surface of other big size of nanoparticles through physical interaction and work as MRI guided delivery 30. In addition, we developed a hybrid system by conjugating a natural enzyme (glucose-oxidase) to dextran-coated iron oxide nanoparticles (similar to ferumoxytol). This strategy resulted in dual catalytic activity whereby the natural enzyme catalyzes glucose (present in biofilms) into H_2_O_2_, and the iron oxide cores catalyze the reaction of H_2_O_2_ in acidic conditions to locally generate ROS that disrupts tooth-decay causing biofilms 31.

## Diagnostic applications

**Figure 2:**
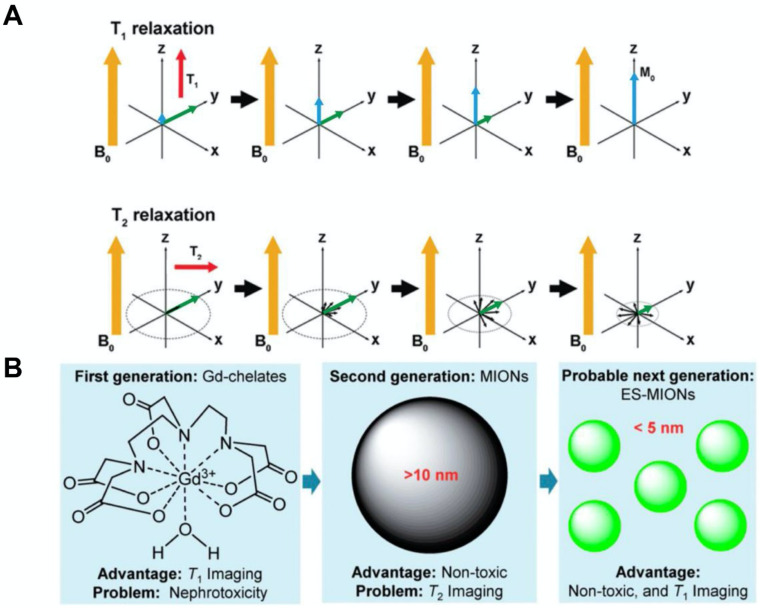
(A) T_1_ relaxation measures the time it takes the initial longitudinal magnetic moment (M_0_) to restore. T_2_ relaxation is a measurement of the reduction of the transverse magnetic moment (M_xy_). (B) A summary of the advantages and disadvantages of first, second and the probable subsequent generations of MRI contrast agents. MIONs: magnetic iron oxide nanoparticles; ES-MIONs: extremely small MIONs. Reproduced with permission from references 43, 48.

MRI is a medical imaging technique that provides high spatial resolution and good soft tissue contrast, but does not expose patients to ionizing radiation. Exogenous magnetic agents have been successfully used to enhance the MRI contrast between healthy tissue and diseased tissue 32-36. MRI contrast agents can affect both the spin-lattice (T_1_) and spin-spin (T_2_) relaxation times of neighboring water protons (Figure 2A). T_1_ refers to the time constant of the recovery process of magnetization along the longitudinal plane after a radiofrequency (RF) pulse. T_1_ relaxation is sensitive to magnetic fields that fluctuate at or about the operational frequency, or the Larmor frequency. T_2_ is the time constant of the decay process of magnetization in the transverse plane after an RF pulse. T_2_ relaxation is mostly determined by low frequency field fluctuations. Low frequency fluctuating fields are more dominant than those at or near the Larmor frequency in biological tissues, and thus, T_2_ values are usually much smaller than T_1_ values. T_2_^*^ further accounts for the dephasing effects caused by extrinsic magnetic field inhomogeneities in addition to intrinsic molecular interactions. T_2_^*^ values are always smaller than T_2_ values in tissues since microscopic magnetic field distributions can be generated by compartmental differences in magnetic susceptibility (*e.g.*, areas surrounding blood vessels). Furthermore, longitudinal relaxivity (r_1_), transverse relaxivity (r_2_), and relaxivity ratio (r_2_/r_1_) are important parameters that determine the imaging characteristics of a MRI contrast agent. The value of r_1_ indicates the signal enhancement potential of an agent, whereas the relaxivity ratio reveals the suitability of an agent for T_1_- or T_2_-weighted MRI. In general, an agent with a lower relaxivity ratio (<5) should provide good T_1_-weighted contrast, while an agent with a higher relaxivity ratio (>10) should be used for T_2_-weighted contrast 37. Currently, most of the commercial MRI contrast agents are gadolinium-based contrast agents (GBCAs). The paramagnetic nature of gadolinium ions shortens the T_1_ relaxation time of nearby protons and increases the signal intensity, resulting in positive contrast enhancement. T_1_-weighted images are ideal for showing detailed anatomical structures. At clinically relevant magnetic fields, GBCAs have similar longitudinal (r_1_) and transverse (r_2_) relaxivities. However, recent reports have indicated that GBCAs may cause nephrotoxicity in patients with insufficient renal function 38, 39. Moreover, GBCAs have been found to deposit in neural tissues and remain there for a long time, with unknown health effects 40, 41.

Compared to GBCAs, iron oxide nanoparticles are safer and more biocompatible since iron is an essential mineral for blood production and other functions (whereas gadolinium is a heavy metal that our bodies are not naturally exposed to). Superparamagnetic iron oxide nanoparticles generate large heterogeneous field gradients when subjected to an external magnetic field. The large magnetic moment causes efficient spin dephasing and alters the T_2_ or T_2_^*^ relaxation time of surrounding protons 42, 43. This leads to a decrease in signal intensity, thus producing images with negative contrast enhancement. T_2_-weighted images provide good pathological information, where abnormal lesions would appear dark due to the presence of these nanoparticles. At clinically relevant magnetic fields, superparamagnetic iron oxide nanoparticles have much greater transverse relaxivities compared to longitudinal relaxivities. In general, r_2_ increases linearly with magnetic strength, while r_1_ decreases slightly 44. Nevertheless, these nanoparticles show superior T_1_-weighted MR enhancement at lower magnetic field strengths 45. Therefore, the potential for mixed weighting effects increases with magnetic field strength in iron oxide nanoparticle-based MRI applications 46. Furthermore, the core diameter of an iron oxide nanoparticle can determine its T_1_ and T_2_ shortening effects (Figure 2B). Large iron oxide nanoparticles generally function as T_2_ agents and have limited use in T_1_-weighted imaging due to high relaxivity ratio (r_2_/r_1_).

On the other hand, ultrasmall iron oxide nanoparticles with core sizes less than 10 nm can more effectively reduce T_1_ relaxation times and enable T_1_ weighted contrast detection, but at the expense of their T_2_ shortening capability 47, 48. Although ferumoxytol nanoparticles possess an ultrasmall core, their large carbohydrate coating shell allows them to bypass kidney filtration, resulting in prolonged intravascular residence time and longer lasting contrast, which can be beneficial for imaging the blood pool and tumors 21, 49, 50. Ferumoxytol may be ideal for use in patients with compromised urinary functions since there is a low likelihood for iron to induce nephrotoxicity, unlike gadolinium or other rare earth metals 51. Interestingly, ferumoxytol can be used as both a positive and negative MR contrast material due to its inherent T_1_ shortening properties and strong T_2_^*^ susceptibility effect. Conventional pulse sequences and delayed MRI can be applied to illustrate MR signal enhancement or loss. This nanoparticle was found to extravasate via discontinuous microvessels and produce higher T_1_ enhancement of tissue in the RES and tumor sites within few hours after administration compared to GBCAs 52, 53. It can increase both T_1_ and T_2_ relaxation by 10-20 fold when compared with an equivalent dose of GBCAs. For example, at 0.5 T, the r_1_ and r_2_ relaxivities of ferumoxytol are 38 and 83 mM^-1^s^-1^, respectively, while those of GBCAs are only 3.8 and 4.1 mM^-1^s^-1^, respectively 54. At 1.5 T, the r_1_ and r_2_ relaxivities of ferumoxytol are 15 and 89 mM^-1^s^-1^, respectively, while those of GBCAs increase slightly 24. Thus, the r_1_/r_2_ ratio generally decreases with increasing magnetic field strengths and is especially pronounced for ferumoxytol nanoparticles 55. For example, using a rapid T_1_-weighted sequence (turbo spin echo), ferumoxytol was found to provide greater signal enhancement at 1.5 T than at 3 T in patients with neural malignancies, while no T_1_-weighted enhancement was found at 12 T 56. Here, we will highlight examples where ferumoxytol is used as a diagnostic imaging agent.

### Stem cell tracking via MRI

Cell imaging is an important tool in the development of cell therapies. Ferumoxytol holds great promise as an MR cell tracking agent due to its biocompatibility and biodegradability 57, 58. Different cell types, such as stem cells and macrophages, have been labeled with ferumoxytol and tracked by MRI for weeks to months after transplantation to monitor cell migration and rejection response 59-61. In particular, mesenchymal stem cells have been a common target for ferumoxytol labeling 62-68. Castaneda et al. described a protocol to label human mesenchymal stem cells, human embryonic kidney 293 (HEK293) cells, and induced pluripotent stem cells using protamine sulfate as a transfection agent to facilitate phagocytic uptake of ferumoxytol 69. They found that as few as 10,000 ferumoxytol-labeled cells could be detected via T_2_-weighted MRI. Khurana et al. labeled adipose-derived stem cells with ferumoxytol using the same protocol and implanted them in rat femurs with osteochondral defects. These labeled cells significantly shortened T_2_ relaxation times compared to unlabeled cells. When tracked by T_2_-weighted MRI, they found that the signal-to-noise ratio (SNR) of labeled cells fell back to baseline at four weeks post transplantation 70.

**Figure 3:**
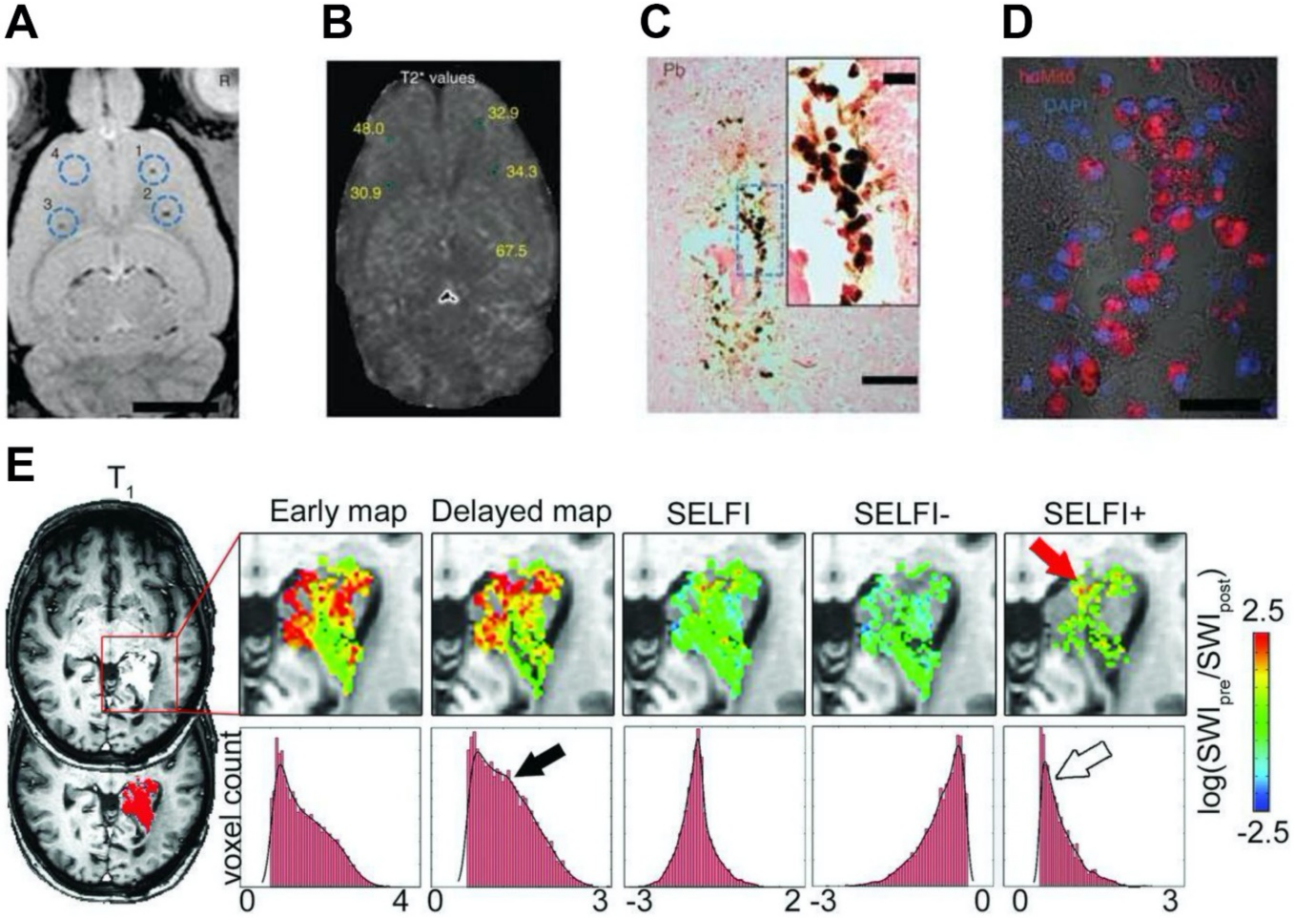
(A) *In vivo* MRI of the brain of a rat injected with differing numbers (10^3^-10^4^) of HPF-labeled bone marrow stromal cells (BMSCs) at locations 1-3 or 10^4^ unlabeled BMSCs at location 4. (B) T_2_^*^ map with the T_2_^*^ values marked for the injection locations relevant to the slice shown in (A). (C) 3,3-diaminobenzidine-enhanced Prussian blue enhanced micrograph of the injection site after administration of 10^3^ HPF-labeled BMSCs. (D) Confocal image of a consecutive tissue section stained by anti-human mitochondrial antibody immunofluorescence. (E) MRI where ferumoxytol was used to detect the macrophage content in glioblastoma. Reproduced with permission from references 71, 85.

Furthermore, Thu et al. used a combination of heparin, protamine sulfate, and ferumoxytol (HPF) to label neural stem cells and track them through serial MRI scans 71. Compared to ferumoxytol alone, HPF increased the T_2_ relaxivity by three folds. They observed as few as 1000 HPF-labeled cells on T_2_^*^-weighted MRI at eight days after intra-cerebral implantation (Figure 3A, 3B). Moreover, the MR signal arose from the labeled cells was corroborated via histology and immunofluorescence (Figure 3C, 3D). Notably, this nanocomplex is comprised of three components that are already approved by the FDA, which may provide the basis for rapid clinical translation of magnetic cell labeling and MRI cell tracking technologies. Yin et al. also used HPF to label human adipose-derived stem cells 72. A small number of labeled cells (10,000) was transplanted into the brains of a rat model of stroke and was visualized by T_2_-weighted MRI. This cell labeling procedure was further optimized by Gutova et al. 73. They determined the optimal ratios of HPF and amount of iron needed to label neural stem cells successfully. Using T_2_-weighted MRI, they detected hypointense regions in the brain at four days after intravenous injection of HPF-labeled cells. At doses higher than expected for human use, no iron-associated safety issues were observed at mid-term (4 weeks) and long-term (12 weeks) after intracranial administration.

Moreover, ferumoxytol can be injected before transplantation to preload macrophages in the RES, then further utilized to monitor differential migration of macrophages into stem cell transplants 59. Daldrup-Link et al. made use of this technique to endogenously label green fluorescent protein expressing macrophages with rhodamine-conjugated ferumoxytol *in vivo* and monitor the migration of labeled macrophages into mesenchymal stem cell transplants in osteochondral defects of female rats and calvarial defects of recipient mice using MRI and intravital microscopy 74. Compared to immune-matched stem cell implants, ferumoxytol greatly enhanced the T_2_-weighted contrast of immune-mismatched stem cell implants, indicating obvious recruitment of labeled macrophages into these transplants. Ferumoxytol was likely taken up by tissue resident macrophages from adjacent bone marrow, spleen, or liver; however, their specific origin could not be determined. Following this work, they conjugated caspase-3 cleavable, fluorescent peptide onto the surface of ferumoxytol and labeled mesenchymal stem cells prior to transplantation 75. When exposed to apoptotic cells, the peptide conjugate was cleaved in the presence of activated caspase-3, releasing fluorescent dye molecules. Thus, this imaging probe can be used for tracking of stem cells via T_2_-weighted MRI and diagnosis of immune rejection-induced apoptosis at an early stage via longitudinal fluorescence imaging.

Additionally, tissue-specific stem cells were also investigated. These stem cells live in specific tissues or organs, and can generate different cell types for where they live. For example, Vandergriff et al. used magnetic targeting to improve the engraftment of transplanted cells 76. They labeled human cardiosphere-derived stem cells with HPF. Then, they delivered the labeled cells by targeting them into a rat heart with myocardial infarction using a magnet. They observed cardiac homing of the transplanted cells as well as increased long term cell retention in the cardiac tissue through monitoring with MRI. Lastly, several studies have focused on labeling and tracking of progenitor cells, which are the early descendants of stem cells. For example, Lamanna et al. labeled human neural progenitor cells with ferumoxytol and injected these cells into the spinal cord of healthy minipigs 77, 78. The labeled cells were tracked by T_2_^*^-weighted MRI for up to 105 days after transplantation. They confirmed that the labeled cells retained the nanoparticles and long term cell survival was not affected by ferumoxytol labeling. They also demonstrated that MRI has the capability to evaluate the accuracy of labeled cell engraftment *in vivo*. In addition, Skelton et al. labeled cardiac progenitor cells with ferumoxytol and transplanted them intramyocardially into healthy pig hearts. The localization and dispersion of these labeled cells could be tracked by T_2_^*^-weighted MRI for up to 40 days 79.

Recent studies have made remarkable progress in developing protocols for labeling stem cells with ferumoxytol as well as establishing MRI as an informative and reliable cell tracking modality. Direct monitoring using cell tracking techniques provides key information on transplanted cells, such as their localization and migration, which can enhance the delivery accuracy and therapeutic outcomes of cell-based therapies. However, more work should be done to improve the long-term viability of labeled cells to allow better quantification and monitoring of the cell of interest. For long term cell tracking, methods to maintain cell retention and prevent signal loss due to exocytosis and cell division should be further investigated. Thorough assessments of delivered dose and safety are needed prior to clinical translation.

### MRI of the central nervous system

The use of ferumoxytol for imaging the physiology and pathology of the central nervous system with MRI has been extensively investigated 80. For example, contrast-enhanced MRI using ferumoxytol can be used for the detection of brain tumors and cerebral aneurysm 81-84. Ferumoxytol can slowly traverse the disrupted blood-brain barrier around the lesions, which results in peak MRI signal changes around 24 hours. These nanoparticles can be largely taken up by tumor-associated macrophages, producing strong negative contrast enhancement on T_2_-weighted images. Notably, ferumoxytol may still be observed in brain pathologies for several days after administration 56. Interestingly, when comparing the performance between GBCAs and ferumoxytol, no significant difference in the MR contrast of metastatic brain lesions was observed between the two agents 21. Furthermore, the ability of ferumoxytol to serve as both delayed T_1_ and T_2_ MR contrast agents may help to differentiate intracellular iron from those in the extracellular space 54. Barajas et al. developed a novel technique named segregation and extravascular localization of ferumoxytol imaging (SELFI) to differentiate MR contrast signals between extravascular and intravascular ferumoxytol pools within treated glioblastoma at a 24-hour delayed imaging time point (Figure 3E) 85. SELFI was optimized based on previous techniques by removing intrinsic tissue and intravascular signal, as well as serving as a more precise imaging metric of glioblastoma-associated macrophage accumulation.

Moreover, macrophage mediated neuroinflammation in brain lesions can be visualized via delayed time point MRI. McConnell et al. demonstrated that ferumoxytol can enhance neuroinflamed regions from surrounding normal brain tissues since most of the nanoparticles were taken up by astrocytes and proinflammatory macrophages, but not by glioma cells 86. Ferumoxytol has also been used to quantify macrophages in high-grade gliomas and to detect active inflammation sites in the central nervous system, such as cavernomas, through susceptibility-weighted MRI 87-89. Importantly, it was found that single and multiple ferumoxytol administrations do not lead to iron deposition in the deep gray structures of the brain of children and young adults with arteriovenous malformations, underscoring the potential of ferumoxytol as a safe alternative for GBCAs 90. Additionally, after brain tumor treatment with chemotherapy or radiation therapy, true tumor progression (tumor size which indicates failure of ongoing therapy) and pseudoprogression can be represented by MR contrast enhancement 91. In the case of pseudoprogression, the change in MRI signal indicates an increase in blood-brain barrier permeation and changes in inflammatory state that are caused by adjuvant treatments. These changes are correlated with a favorable prognosis 92.

Ferumoxytol nanoparticles have shown to be a suitable contrast agent for MRI of central nervous system pathologies, such as vascular malformations and glioblastoma. The ability of ferumoxytol to assess and quantify the inflammatory component (*i.e.*, macrophages) of brain tumors has also been demonstrated. The iron oxide cores can be further metabolized by the human body, thus minimizing possible health risks from long term retention of iron in neural tissues. However, there are no studies that assess the long-term deposition of ferumoxytol in the human brain thus far. Therefore, future investigations will have to determine safety and clearance of iron in patients with impaired metabolic functions. Furthermore, factors, such as repeated injections and iron doses, that may lead to significant iron deposition in the brain will have to be assessed.

### MRI of the lymphatic system

As previously mentioned, ferumoxytol can be taken up by macrophages in the tumor microenvironment as well as those in the RES. This allows ferumoxytol to be utilized as a lymphotrophic agent for imaging and mapping of benign and metastatic lymph nodes throughout the body. Thus, ferumoxytol has been investigated for staging and nodal evaluation via MRI in various malignancies, such as prostate and pancreatic cancers. It was determined that a dose as low as 0.15 mg Fe/kg can be used for lymph node visualization 93. The optimal dose of ferumoxytol for lymph node visualization was further studied in patients with prostate cancer using 3 T MRI 94. This phase I trial recruited patients who underwent radical prostatectomy along with bilateral pelvic lymph node dissection. Patients were imaged via abdominopelvic MR lymphography at 3 T before and 24 hours after ferumoxytol administration using T_2_- and T_2_^*^-weighted sequences. It was shown that 7.5 mg Fe/kg ferumoxytol was effective and safe for enhanced imaging of benign lymph nodes. Furthermore, the detection of metastatic lymph nodes was evaluated in patients with prostate, bladder, and kidney cancer in phase II trial studies 95. MR lymphography using ferumoxytol yielded high detection sensitivity of metastatic lymph nodes in these cancer types. There is no difference in contrast enhancement between benign and malignant lymph nodes at 24 and 48 hours, indicating that imaging can be achieved safely within 24 or 48 hours of ferumoxytol administration. In addition, McDermott et al. investigated metastatic nodal involvement preoperatively in patients with pancreatic ductal adenocarcinoma via T_2_^*^-weighted, ferumoxytol-enhanced MRI 96. Compared to conventional MRI, this imaging method allowed malignant lymph nodes to be identified on a node-by-node basis with much higher accuracy (0-42% for conventional MRI vs. 76% for contrast enhanced MRI), specificity, and detection sensitivity. Therefore, ferumoxytol-enhanced MRI can safely and accurately determine nodal status in several primary tumors, as well as improve preoperative management options. Additionally, the use of ferumoxytol for lymph node detection was studied in pediatric patients since the cellular composition of benign lymph nodes in children is different from that in adults. It was found that pediatric patients have less lymphocytes and more vascularized lymph nodes compared to adult patients 97, 98.

Ferumoxytol has performed well as a lymphotrophic nanoparticle that can identify metastatic nodes and stage them on a nodal basis via MRI. However, the efficacy of this agent still needs to be validated in larger trials since most of the current clinical findings are based on limited patient samples. In addition, specificity of ferumoxytol-enhanced MRI might be biased due to the small number of malignant lymph nodes.

### MRI of the vascular system

Ferumoxytol has recently been applied in both pediatric and adult patients for vascular MRI of various parts of the body, such as the chest and abdomen 99. As previously mentioned, ferumoxytol has a prolonged blood residence time of approximately 14 hours due to its size and coating. This long intravascular half-life extends the vascular signal, which is ideal for imaging the blood pool, and allows for repeated and delayed imaging without additional injections of contrast material 100. Ferumoxytol administration has shown hyperintense intravascular enhancement on T_1_-weighted images without causing any nephrotoxicity or nephrogenic issues 101. In addition, ferumoxytol can be phagocytosed by macrophages after a delay, which enables the imaging of inflammation in the vessel wall or macrophage burden in atherosclerotic plaques 102. Li et al. evaluated the feasibility of first-pass ferumoxytol-enhanced MR angiography (MRA) in humans 103. MRA is generally preferred in younger patients since this technique does not utilize ionizing radiation. They examined different vessels including carotid arteries, peripheral arteries, thoracic aorta, and abdominal aorta. First-pass ferumoxytol-enhanced MRA demonstrated prolonged steady-state arterial enhancement and selective venous enhancement on delayed acquisitions, which allows arteries and veins to be depicted in a single imaging exam. Wilson et al. found that ferumoxytol has the potential to conduct 3-D MRA of all abdominal vessels in a single breath-hold acquisition 104.

Furthermore, Nguyen et al. showed the possibility of ferumoxytol-enhanced MRA for vascular mapping in renally impaired patients who underwent transcatheter aortic valve replacement 105. In this study, ferumoxytol administration could minimize the dose of iodine-based contrast material used, thereby reducing the potential risk of acute nephropathy. In addition, Walker et al. assessed the feasibility of ferumoxytol-enhanced MRA in patients with peripheral artery disease (PAD), who typically have a higher incidence of renal insufficiency than the general population 106. This pilot study was performed using acquisition parameters similar to those used for imaging GBCAs. The resulting images demonstrated good depiction of suspected lower extremity vasculature and were consistent with MRA performed with GBCAs. Thus, this technique can effectively avoid risk of contrast related complications and be used by vascular surgeons for making clinical decisions in patients with PAD. Stoumpos et al. compared MRA for vascular mapping between ferumoxytol and duplex US 107. Ferumoxytol-enhanced MRA can identify central vessel pathologies and PAD, which could not be recognized by duplex US. This suggested that ferumoxytol was more predictive than duplex US when considering the outcome of arteriovenous fistula surgery. Lastly, Yilmaz et al. demonstrated that infiltrating macrophages in injured myocardium can be detected by ferumoxytol via T_2_^*^-weighted MRI, thereby providing more detailed characterization of myocardial infarct pathology 108.

Ferumoxytol has been shown to provide strong vascular contrast over a long period of time. This feature has been exploited for venous imaging, repeated imaging, and delayed imaging. These imaging sequences may be beneficial for evaluating key vascular regions, such as aorta and lower extremity arteries. Future studies will have to include larger patient samples and determine the optimal dosing strategy of ferumoxytol for vascular imaging with MRA in children, as well as those with compromised renal functions. The systemic side effects of iron will have to be thoroughly assessed, especially in pediatric patients.

### Combination of MRI and other imaging modalities

**Figure 4:**
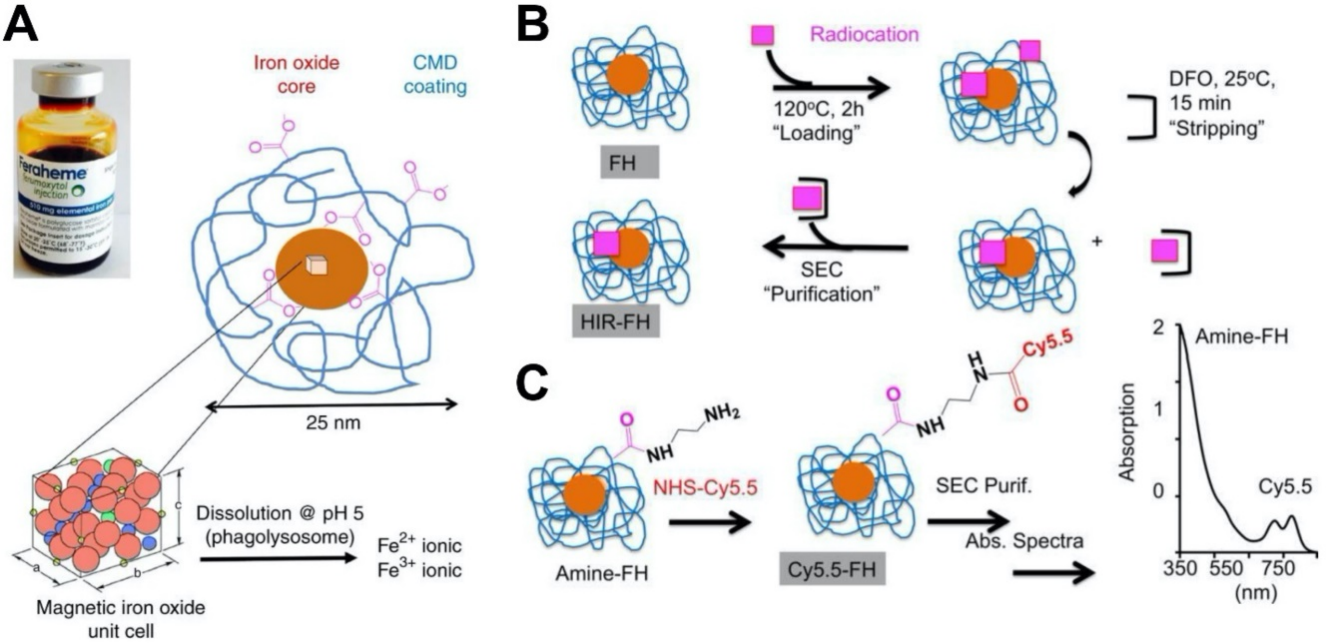
(A) Ferumoxytol consists of a superparamagnetic iron oxide core surrounded by carboxymethyl dextran (CMD) coating that can be exploited to incorporate additional contrast generating materials. (B) Heat-induced radiolabeling of ferumoxytol. A radiocation (^64^Cu^2+^, ^111^In^3+^ or ^89^Zr^4+^) is heated with ferumoxytol during the 'loading' phase, then purified via incubation with deferoxamine mesylate followed by size-exclusion chromatography. (C) The carboxyl groups of dextran within ferumoxytol can be reacted with ethylene diamine to obtain amine-ferumoxytol, which can then be reacted with the NHS ester group of Cy5.5 as evidenced by spectrophotometry. Reproduced with permission from reference 28.

Integration of multiple imaging modalities has been used in numerous preclinical and clinical settings for disease detection and other applications. Multi-modal imaging can combine various techniques, such as positron emission tomography (PET), MRI, computed tomography, and magnetic resonance spectroscopy. For examples, PET/MR imaging can be beneficial for attenuation correction (AC) of PET data based on MRI information that has been processed into an MR-derived attenuation map (mu-map), thus enabling the quantification of PET information 109. Consequently, there has been interest in combing ferumoxytol with other contrast generating materials to form novel nanohybrids for multimodality imaging. Yuan et al. developed ferumoxytol nanocomposites for PET or single-photon emission computed tomography (SPECT) imaging using a heat-induced radiolabeling method (Figure 4) 28. Radioisotope cations that are widely used in clinical settings, including ^89^Zr^4+^ or ^64^Cu^2 +^ for PET and ^111^In^3 +^ for SPECT, were attached to ferumoxytol through a chelateless radiocation surface adsorption method. In addition, ferumoxytol could be rendered fluorescent by conjugation of Cy5.5 via a diamine linker. The radiocations have minimal influence on the physical and biological properties of the ferumoxytol 110. It was found that the accuracy of PET measurements can be influenced by MR-related temporal changes when PET imaging and MRI are conducted simultaneously 109. Moreover, a single dose of ferumoxytol can decrease the uptake values of 2-deoxy-2-[^18^F] fluoro-D-glucose (^18^F-FDG) up to 53% when whole-body ^18^F-FDG PET imaging was performed with MRI 111. This finding suggests that MRI based AC is needed prior to the administration of the hybrid agent. Following this work, Muehe et al. investigated whether the standardized uptake values in normal organs can be changed by the administration of ferumoxytol prior to ^18^F-FDG PET/MRI scan 112. They studied 613 lymph nodes from children with cancer. They found a marked hypointense hilum in benign lymph nodes on T_2_-weighted scans, which indicated that ferumoxytol enhancement patterns of benign lymph nodes in children were different from adult patients. The distribution of ferumoxytol nanoparticle at the hilum can be applied to diagnose a benign lymph node for pediatric patients. It was shown that this administration would not change standardized uptake values of solid extra-cerebral organs. Thus, ferumoxytol administration prior to a PET/MRI procedure was used for accelerating the image acquisition times. Additionally, ferumoxytol was also used to image lymph nodes in pediatric cancer patients via ^18^F-FDG PET/MRI 113. A difference in nodal patterns between children and adults was observed from ferumoxytol-enhanced MRI, underscoring the ability of benign lymph nodes to be diagnosed by ferumoxytol accumulation in the hilum.

In future studies, we expect to see more progress made in engineering ferumoxytol nanohybrids with other radiotracers (or radiocations) and contrast generating materials to enable imaging of additional diseases via different modality combinations.

## Therapeutic Applications

### Iron deficiency treatment

Iron is critical for many physiological processes in the body. Low levels of iron can lead to anemia, which currently affects 1.2 billion people in the world and is the most prevalent nutritional deficiency 114. IDA occurs when the iron content in the body is low or when iron is withheld from serum plasma as a result of ongoing inflammation 115. This health issue is common in young children, pregnant women or patients with inflammation. IDA is traditionally treated by oral iron supplements, however, oral iron agents are often ineffective due to gastrointestinal side effects, poor adherence, and impaired absorption 116, 117. Therefore, intravenous iron therapy serves as an alternative to oral iron therapy since it can increase hemoglobin levels, and reduce the dose of erythropoiesis stimulating agents 118. Ideally, intravenous iron agents should have low adverse effects, ease of administration, and low free-iron toxicity 19. Currently, there are several intravenous iron agents developed for iron therapies, including ferumoxytol, ferric isomaltoside, and ferric carboxymaltose, which are detailed in Table 2 119-122. All of them consist of an iron core that is coated and stabilized by a shell of carbohydrates. The surrounding carbohydrates play an important role in slowing the release of iron *in vivo* and keeping the iron oxide nanoparticles in a colloidal suspension 123. After intravenous administration, the complexed iron is taken up by phagocytic cells in the RES organs, which is then incorporated into ferritin. These iron-containing proteins could be stored intracellularly or could be bound to transferrin and delivered as erythroid precursors for subsequent hemoglobin synthesis.

In clinical settings, ferumoxytol is intravenously injected at an infusion rate of 30 mg per ml per second (or 510 mg in 17 ml for a duration of seventeen seconds) 19, 20. The same iron dose may be administered again approximately 3-8 days after the first injection. Compared with oral iron agents, ferumoxytol was reported to be more effective and well tolerated in patients with IDA. Provenzano et al. enrolled 230 IDA patients and administered ferumoxytol to one half of these patients and oral irons to the other half 124. IDA patients who received two doses of ferumoxytol (administered one week apart) showed higher levels of hemoglobin compared to those who received oral irons. Ferumoxytol was well tolerated in these patients with no significant occurrence of anaphylaxis. Additionally, in a phase III study, it was found that IDA patients who were treated with ferumoxytol have increased hemoglobin levels and feel more energetic and less fatigued 125.

Since the composition of ferumoxytol is similar to other intravenous iron agents, a direct comparison of ferumoxytol with other iron formulations has been conducted. Ferumoxytol exhibits tighter iron binding and lower amounts of labile free iron when compared to iron agents, such as iron sucrose, ferric gluconate, and iron dextran. These properties allow ferumoxytol to be administered at a much higher dose. Macdougall et al. compared the safety profiles between ferumoxytol and iron sucrose in patients with chronic kidney disease 126. It was shown that the SAE profiles between the two formulations were quite similar. In addition, the hemoglobin concentration increased in a similar manner between the two treatment groups. According to this result, ferumoxytol and iron sucrose have equivalent efficacy and SAE rates. Adkinson el al. also compared the efficacy between ferumoxytol and ferric carboxymaltose for the treatment of IDA 118, 127. The patients were administered either ferumoxytol (510 mg) or ferric carboxymaltose (750 mg), and each iron agent was administrated on days 1 and 8 or 9. These two iron formulations demonstrated comparable efficacy in raising hemoglobin levels despite a difference in the iron dose.

When treating IDA patients with ferumoxytol, safety is always an important consideration. This has prompted many investigations through clinical trials. For example, Singh et al. recruited patients with chronic kidney disease, and about half of them were given ferumoxytol, while the other half was given placebo 128. During this study, 420 adverse events were reported (*i.e.*, 21.3% with ferumoxytol and 16.7% with placebo). The most observed adverse events were dizziness, pruritus, headache, fatigue, and nausea. Only 0.1% of the patients who had ferumoxytol reported more serious adverse events including anaphylaxis. It was demonstrated that ferumoxytol is well tolerated and can be administrated safely in anemic patients with impaired renal functions. However, it is worth noting that there were several post-marketing reports of SAEs stemming from the use of ferumoxytol 129. As a consequence, ferumoxytol was removed from the European market in 2015 114. The FDA and Health Canada then increased the infusion duration from 17 seconds to 15 minutes to reduce the frequency of hypersensitivity reactions 130. Overall, based on the preponderance of published evidence, ferumoxytol is safe for the treatment of IDA in the presence of renal insufficiency. Importantly, the safe use of ferumoxytol for IDA treatment in pregnant women was reported recently 131. In the future, more research will be done to assess the safety and efficacy of ferumoxytol for treating IDA in pediatric patients.

### Treatment based on ROS generation

Iron has crucial roles in supporting physiological functions such as oxygen transport through hemoglobin, which is an essential process associated with respiration, DNA repair, and DNA synthesis 132. However, under certain circumstances, iron can also trigger oxidative stress based on its pro-oxidative capability for treatments. Among them, one of these treatments is based on ferroptosis. First, intracellular iron can generate a large amount of ROS via the Fenton reaction. Then, intercellular ROS can propagate lipid peroxidation burst to promote ferroptosis that results in cellular damages. In addition, intracellular iron levels may influence the activity of other ROS-generating enzymes, such as nicotinamide adenine dinucleotide phosphate oxidases (NOXs), lipoxygenases, and mitochondrial electron transport complexes, resulting in ferroptosis 133-136. Thus, this mechanism induced by iron can be applied to immunotherapy as well as anti-leukaemia, which we describe below.

#### Ferumoxytol-based immunotherapy

**Figure 5:**
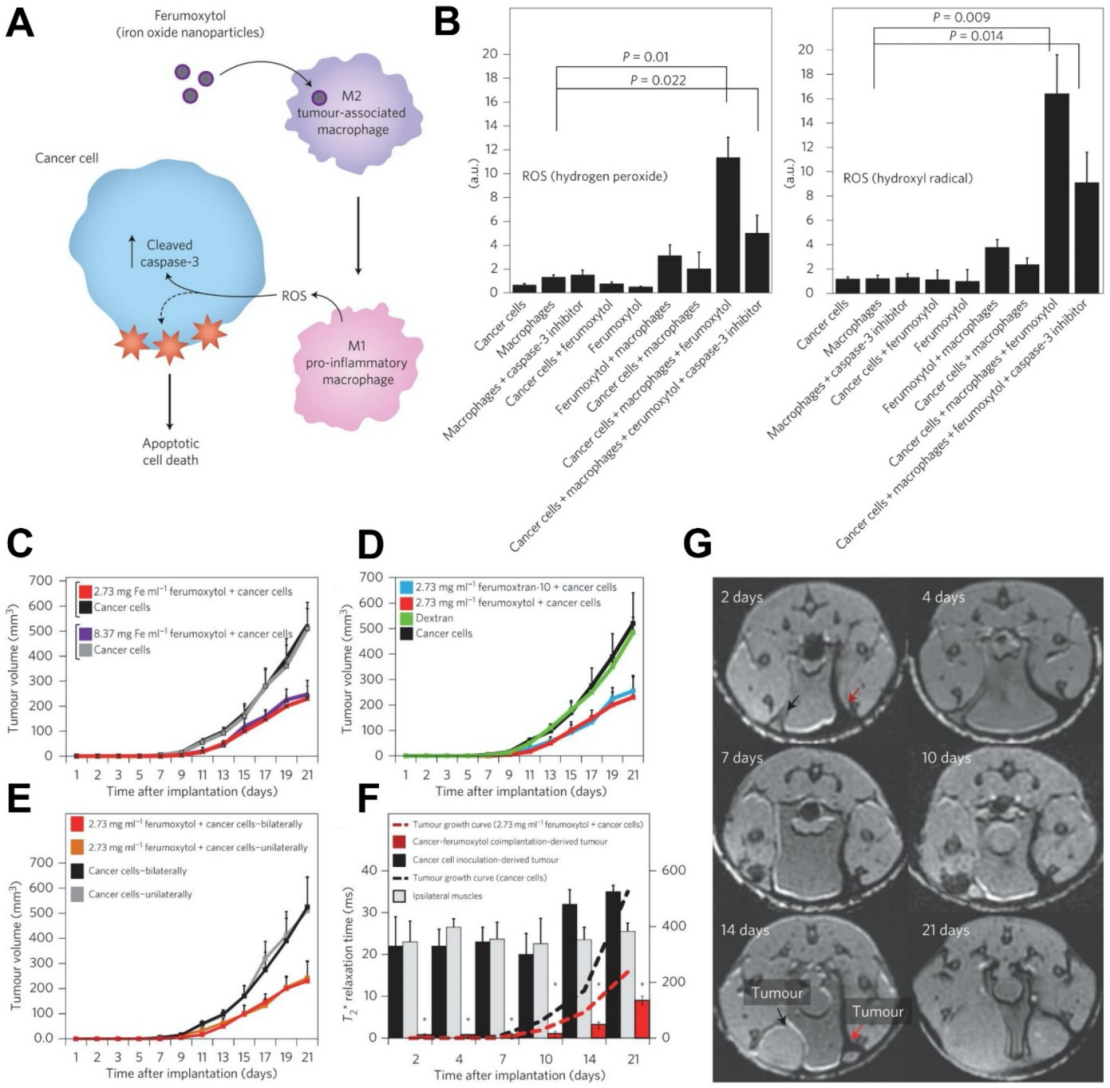
(A) The mechanism of cancer cell killing using ferumoxytol through altering the polarization of tumor associated macrophages. (B) Quantification of hydrogen peroxide (left) and hydroxyl radicals (right), which indicating their heightened levels in macrophage, cancer cell and ferumoxytol co-cultures. (C) Various concentrations of ferumoxytol suppressed tumor growth when compared with untreated controls. (D) Tumor growth was significantly inhibited by two types of iron formulations (ferumoxytol and ferumoxytran-10) while dextran alone did not. (E) To exclude cross-talk of two tumors in the same mouse, tumor growth was compared for cancer cells that were inoculated in the mammary fat pad unilaterally and bilaterally, with no significant difference in tumor volume being observed. (F) Analysis of MRI of cancer inoculation sites treated and untreated with ferumoxytol. Paraspinal muscle data is included as internal control. (G) Corresponding T_2_^*^-weighted MRI of a mouse inoculated with cancer cells with (right, red arrow) or without (left, black arrow) ferumoxytol. Reproduced with permission from references 140, 173.

Iron oxide nanoparticles have various effects on the immune system 137. Many preclinical studies and clinical trials have indicated that iron oxide nanoparticles can interact with immune cells and improve cancer treatments through immunomodulatory activities 138-140. Iron oxide nanoparticles have been found to stimulate a 'pro-inflammatory' immune cell phenotype in macrophages 140-142. Zanganeh et al. investigated ferumoxytol therapy on early mammary cancers and lung cancer metastases 140 (Figure 5). Hydrogen peroxide and hydroxyl radicals were elevated in co-cultures of macrophages, cancer cells and ferumoxytol (Figure 5B). It was shown that adenocarcinoma cells caspase-3 activity was enhanced after incubation with ferumoxytol-containing macrophages. In addition, the macrophages exhibited increased mRNA associated with pro-inflammatory Th1-type responses after incubation with ferumoxytol. Furthermore, the growth of subcutaneous adenocarcinomas in mice was significantly inhibited by ferumoxytol (Figure 5C-5E) and accompanied by an enhanced presence of pro-inflammatory M1 macrophages in the tumor tissues. This therapeutic effect of ferumoxytol on cancer growth was achieved by inducing tumor-associated macrophage (TAM) polarization into the pro-inflammatory M1 phenotype. These macrophages release hydrogen peroxide, which subsequently is converted to ROS by iron to result in cancer cell cytotoxicity. In addition, the MRI contrasting properties of ferumoxytol allowed its metabolism in the tumors to be tracked over time (Figure 5F, 5G). Therefore, ferumoxytol can trigger ferroptosis in cancer cells by inducing TAM transformation to the M1 subtype.

Ferumoxytol was also combined with a toll-like receptor 3 (TLR3) agonist to activate macrophages for halting melanoma growth 143. The TLR3 agonist, or polyinosinic-polycytidylic acid (PIC), was exploited for cancer treatment due to its potential capability to enhance immune system activity 144, 145. Ferumoxytol not only protects PIC from degradation, but also amplifies the immune-stimulatory properties of macrophages for the treatment of both subcutaneous and pulmonary metastasis in melanoma. In another study, the surface of ferumoxytol was modified with β-glucan to form a nanocomposite that regulates macrophage phenotype and produces a synergistic effect on tumor regression 146. This nanocomposite was confirmed to prevent melanoma growth efficiently through stimulating the macrophages into the proinflammatory M1 phenotype. The mechanism for this macrophage polarization induction was mainly associated with the activation of mitogen-activated protein kinase (MAPK) and spleen associated tyrosine kinase (Syk)/nuclear factor kappa-B (NF-κB) signaling pathways.

In addition to stimulating macrophages, ferumoxytol can modulate immune properties in tumors through myeloid-derived suppressor cells (MDSCs). MDSCs are a type of immature myeloid cell that has potent immune suppressive capacity. Xu et al. revealed the immunomodulatory properties of ferumoxytol on MDSCs and determined the mechanism of action 147. In this study, they found that the immunosuppressive function of MDSCs is reduced by ferumoxytol, resulting in significantly lower levels of Arg-1 and ROS. *In vivo* studies showed that lipopolysaccharide induced immunosuppression in the late stage of sepsis can be improved by ferumoxytol due to decreased immunosuppressive properties of T cells and MDSCs.

#### Ferumoxytol-based anti-leukaemia treatments

**Figure 6:**
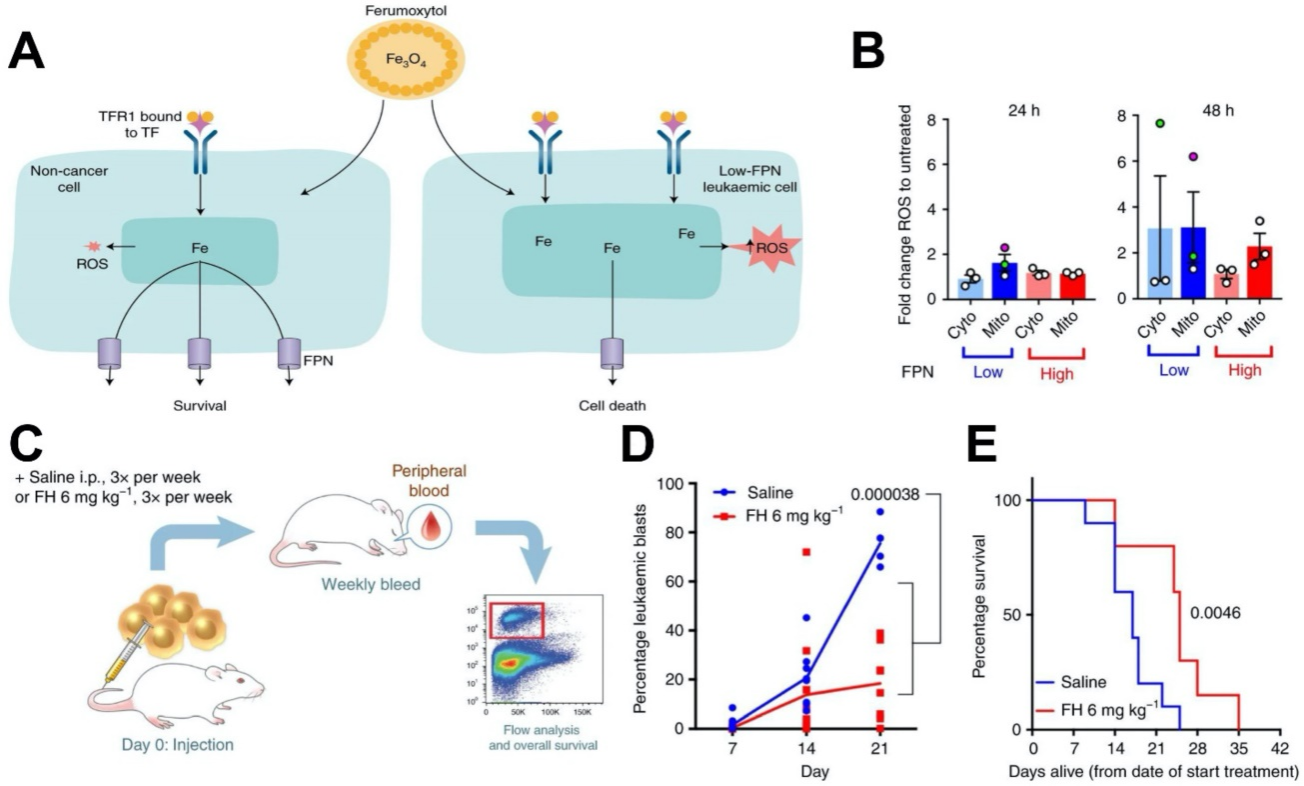
(A) A schematic depiction of how ferumoxytol induces selective cell death for leukaemic cells with low FPN expression. (B) ROS staining in cytoplasm and mitochondria indicate enhanced ROS levels in cell lines with low FPN expression. (C) Schematic depiction of the design of the experiment to investigate the effect of ferumoxytol on the survival rate in a mouse model of leukaemia. (D) Effect of ferumoxytol treatment on leukaemic blast levels. (E) The survival plot for mice administrated using saline or ferumoxytol. Reproduced with permission from reference 149, 150.

Recently, excessive iron has been reported to selectively kill leukaemic cells and spare normal hematopoietic cells 148. Trujillo-Alonso et al. reported that ferumoxytol exhibits anti-leukaemic effects 149, 150 (Figure 6). In this work, they found that ferumoxytol can increase the intracellular iron content in leukaemic cells with low amounts of ferroportin (FPN) *in vitro*, thus enhancing the levels of intracellular ROS (Figure 6B). Then, they investigated the efficacy of ferumoxytol treatment *in vivo* using a murine model of acute leukaemia (Figure 6C-6E). After treating the mice with ferumoxytol, tumor burden was greatly reduced and survival was significantly prolonged. In this case also, the anti-cancer effect of ferumoxytol is considered to be mediated by ROS generation. However, this study relies on the rare naturally occurring low expression of ferroptosis-related genes such as FPN, which may not actively lower the expression of these genes in tumors of patients. Thus, this ferumoxytol-induced ferroptosis therapy may not be broadly applied to other types of cancers.

#### Ferumoxytol-based anti-biofilm treatments

**Figure 7:**
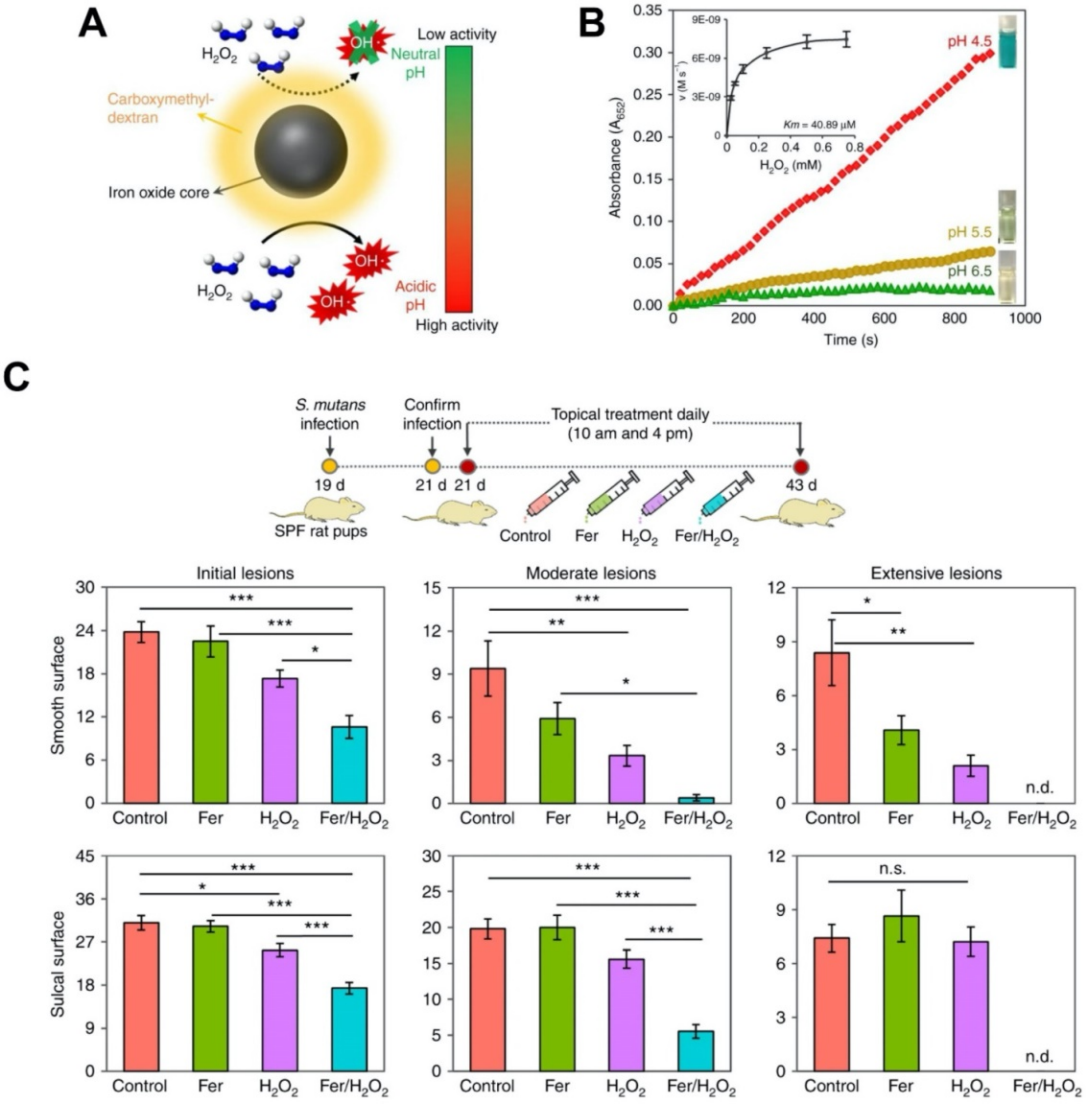
(A) Schematic depiction of ferumoxytol catalytic activity based on different pH values. (B) Catalytic activity of ferumoxytol at various pH values. Inset: Michaelis-Menten constant calculation. (C) Therapeutic effects of ferumoxytol on a biofilm-caused oral disease (dental caries) *in vivo* 174, 175. The graph represents the extent of caries lesions (tooth-decay) on two different dental surfaces (smooth and sulcal surfaces); n.d. means non-detected. Ferumoxytol activation of H_2_O_2_ effectively inhibit the development of tooth-decay, completely preventing cavitation (extensive lesion). Fer: ferumoxytol. Reproduced with permission from reference 13.

In 2007, iron oxide nanoparticles were found to have intrinsic catalytic activity that is similar to horseradish peroxidase (HRP) under acidic conditions 25. Nanoparticles that exhibit high catalytic activity have been termed nanozymes and are considered as an alternative to natural enzymes since they demonstrate better catalytic performance and are lower in cost. Iron oxide-based nanozymes may exhibit more than one type of catalytic activity. They can display catalase-like biocatalytic activity under neutral pH, which can convert hydrogen peroxide into oxygen and water, thus relieving cellular oxidative stress. In addition, they can switch to a peroxidase-like pattern and convert hydrogen peroxide into hydroxyl radicals (OH•) under acidic pH. This is useful for ROS-induced cellular or bacterial killing and has potential to improve ROS-dependent therapies. Therefore, iron oxide-based nanozymes can provide different functionalities based on the target disease applications 151-155. Over the past years, nanozymes have been explored for various therapeutic applications such as tumor prevention, biofilm disruption, anti-oxidation, and so forth as reviewed previously 156-160. Recently, our group has made use of the catalytic activity (peroxidase-like) of iron oxide nanoparticles for treating dental biofilms via activation of hydrogen peroxide and ROS generation. It was found that iron oxide nanoparticles can penetrate and retain within biofilms following topical application, and locally activate hydrogen peroxide to both degrade exopolysaccharides and eliminate bacterial pathogens in the acidic microenvironment within the biofilm 13, 31, 157, 161. Moreover, ferumoxytol can also disrupt pathogenic oral biofilms and prevent tooth decay (dental caries) via similar intrinsic peroxidase-like activity 13 (Figure 7). Notably, the catalytic activity of ferumoxytol is greater at low pH, which provides selectivity for pathogenic biofilms since they create acidic pH microenvironments (Figure 7A, 7B). It was shown that ferumoxytol can localize within the biofilm and produce ROS in the presence of hydrogen peroxide, causing in situ bacterial death and exopolysaccharides degradation. As shown in an *in vivo* study, topical oral treatment with ferumoxytol and hydrogen peroxide can efficiently prevent the onset of severe tooth decay in a rodent model (Figure 7C). In addition, no adverse effects were observed from oral microbiome and histological analyses. The catalytic activity of ferumoxytol has been further evaluated clinically. It was found that ferumoxytol can specifically kill *Streptococcus mutans* (a cariogenic pathogen) via in situ ROS generation and suppress tooth-decay in an intra-oral human disease model. Ferumoxytol has high specificity for *S. mutans* due to preferential binding to specific glucan binding proteins on the bacterial membrane, which allows precise targeting (and localized catalysis) of this pathogen instead of other types of oral bacteria, including commensals. This is the first study that demonstrates the potential therapeutic application of ferumoxytol as anti-infective agent in humans 22.

## Theranostic Applications

**Figure 8:**
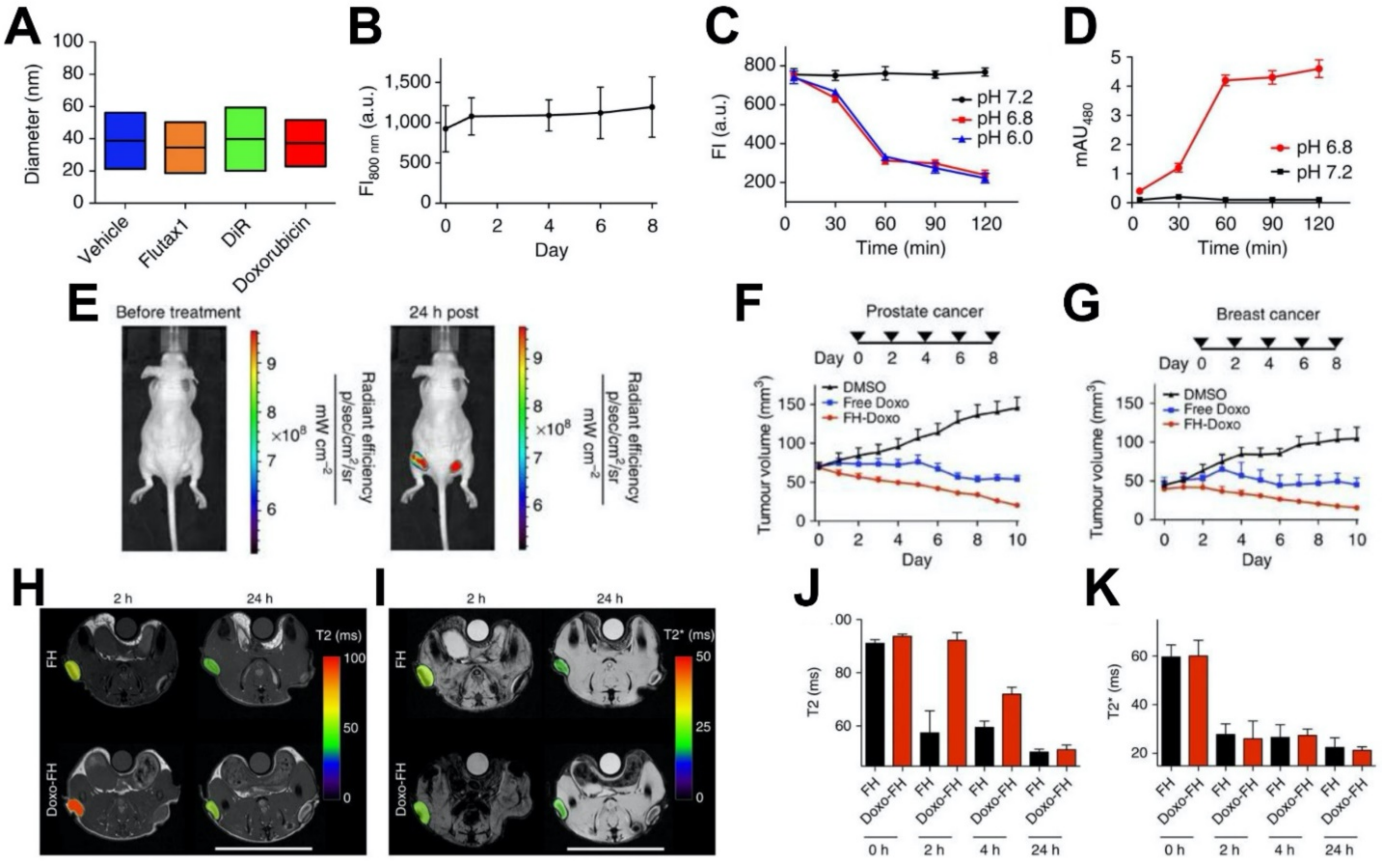
(A) Size distribution of ferumoxytol loaded with the cargoes noted (vehicle is unloaded ferumoxytol). (B) The fluorescence emission of DiR loaded ferumoxytol in fetal bovine serum, with the lack of change indicating its stability. (C, D) Doxorubicin optical signals in (C) an inner chamber containing the nanoparticle-drug complex and (D) an outer chamber containing only media, in different pH conditions. The change in intensity at the lower pH values indicates drug release. (E) Fluorescence imaging of tumor inoculated mice injected with DiR-loaded ferumoxytol indicating tumor accumulation. (F, G) Ferumoxytol loaded with doxorubicin significantly decreased tumor sizes in mice bearing (F) human prostate and (G) human breast xenografts. (H) T_2_ and (I) T_2_^*^ MRI of ferumoxytol and ferumoxytol-doxorubicin injected tumor bearing mice. (J) Quantification of the T_2_ signal allowed the monitoring of drug release over time, since the drug loading affected the transverse relaxivity of ferumoxytol. (K) Quantification of T_2_^*^ values (which are unaffected by drug loading) indicated that there was no difference in the tumor uptake of the unloaded and loaded nanoparticles. Reproduced with permission from reference 29.

In recent years, iron oxide nanoparticles have been applied as a multifunctional nanovehicle that possesses both therapeutic and diagnostic properties 162-164. More specifically, ferumoxytol can act as a carrier for chemotherapeutic drugs and a contrast agent for MRI to monitor the distribution of the therapeutical molecules and provide diagnostic information. For example, Kaittanis et al. utilized ferumoxytol to efficiently deliver various drugs to tumor sites 29 (Figure 8). Small therapeutic molecules can be retained within the carboxymethyl-dextran coating via weak electrostatic interactions and be released under slightly lower pH (Figure 8A-8D). Doxorubicin could be released from ferumoxytol at slight acidic condition (Figure 8C). Treatment with ferumoxytol-drug combinations led to better tumor control than free drug alone, in both breast and prostate cancers (Figure 8F, 8G). Moreover, the drug loading level is proportional to both transverse T_2_ and longitudinal T_1_ nuclear magnetic resonance proton relaxation times. Thus, loading and releasing of drugs can be monitored through the changes in MRI signal intensity (Figure 8H-8K). In an interesting example, Choi et al. developed a theranostic agent that provides MRI-guided immunotherapy of prostate cancer via immune checkpoint blockade 30. The agent, which is consisted of a mesoporous silica nanoparticle loaded with anti-programmed cell death ligand 1 (PD-L1) antibodies and capped with ferumoxytol (Fer-ICB-UPMSNP), was administered following cabazitaxel (Cbz) based chemotherapy. The large pores of the mesoporous silica nanoparticles allowed high loading of PD-L1 antibodies. Ferumoxytol capping not only enabled MRI-guided delivery, but also sustained release of PD-L1 antibodies. Cbz chemotherapy was found to induce immunogenic cell death, promote dendritic cell maturation, and increase PD-L1 level in cancer cells. *In vivo* studies showed that the tumor growth was significantly reduced as a result of sequential MRI-guided local delivery of Fer-ICB-UPMSNP after Cbz treatment. This treatment regime initiated a tumor specific adaptive immune response by effectively activating T cell infiltration and decreasing the number of regulatory T cells. Furthermore, Mohanty et al. used ferumoxytol-based MRI to monitor tumor associated macrophage (TAM) response to CD47 monoclonal antibodies (mAb) therapy in osteosarcomas 165. CD47 mAb can block CD47-SIRPα interactions, thus resulting in the activation of TAMs and phagocytosis of both tumor and ferumoxytol. The uptake of ferumoxytol further enhances T_2_ contrast generation and allows for the monitoring of CD47 mediated immune response. Notably, ferumoxytol-based MRI has distinct advantage over biopsies because it can avoid sampling errors by depicting TAM distribution in the entire tumor. Ferumoxytol-based MRI was also used to quantify TAM in mouse models of anaplastic thyroid cancer 166. The imaging results showed greater uptake of ferumoxytol by TAMs in orthotopic thyroid tumors compared to pulmonary lesions. It was also found that B-Raf kinase inhibitor and PD-L1 antibody combination treatment greatly reduced core TAM accumulation. Recently, ferumoxytol was also explored to detect pathogenic dental biofilms via catalytic activation of hydrogen peroxide in the presence of chromogenic substrate for facile colorimetric visualization 22.

Ferumoxytol has been used as a diagnostic companion for nanoliposomal irinotecan in a clinical pilot study. Nanoliposomal irinotecan is a chemotherapeutic drug that can be delivered to the tumor sites via discontinuous tumor vasculature. Therefore, MRI using ferumoxytol can indicate nanoparticle uptake in tumors prior to nanoliposomal irinotecan treatment. Preliminary data suggest that T_2_^*^ MRI allowed for quantitation of iron particle levels in plasma, reference tissue, and tumor lesions in patients, which can be considered as a predictive biomarker of nanoliposomal irinotecan. It was shown that higher ferumoxytol levels as quantified by MRI were correlated with greater reduction in lesion size, demonstrating the potential clinical application of ferumoxytol as a companion diagnostic 167-169. This approach was further used in a Phase I study to investigate the correlation between ferumoxytol quantitation in tumor lesions and response to nanoliposomal irinotecan in patients with metastatic breast cancer 170.

## Conclusions

Ferumoxytol, in addition to being an FDA approved iron oxide nanoparticle for iron-deficiency anemia treatment, possesses many unique properties and is extensively used in biomedical research. Here, we summarize the developments of ferumoxytol in various biomedical applications including diagnosis, therapy and theranostics (see Table 3 for examples).

First, ferumoxytol is frequently employed as an “off-label” MR contrast agent. Ferumoxytol-based MRI has been used for stem cell tracking and imaging of various pathologies and body structures. Furthermore, ferumoxytol exhibits affinity toward macrophages, which allows for MRI of metastasis in the lymph nodes as well as inflammation in the brain and vasculature. Ferumoxytol has shown to be an effective contrast agent for both T_1_- and T_2_-weighted MRI with minimal long-term retention in neural tissues. In addition, ferumoxytol has a long intravascular half-life and is generally regarded as nontoxic. Therefore, ferumoxytol may be more beneficial than clinically approved gadolinium-based chelates as a potential MR contrast agent. However, long-term deposition of ferumoxytol in patients for MRI still needed to be investigated even though the safety profile was assessed 171, 172. Second, ferumoxytol is an approved intravenous iron formulation for treating IDA patients who have an intolerance to oral iron agents. In particular, several clinical trials have studied the safety and efficacy of ferumoxytol in IDA patients with chronic kidney disease as well as in pediatric population. However, further studies should be done in children with IDA since not much is known about the effects of ferumoxytol on this patient population. Third, ROS generation via iron-based Fenton reaction and from catalytic activation of hydrogen peroxide as enzyme mimics (also termed nanozyme) can be used to treat various diseases. Ferumoxytol can interact with immune cells and alter the polarization of tumor related macrophages to 'pro-inflammatory' immune cell phenotypes. In turn, ferumoxytol increases the production of ROS, which results in oxidative damage to cancer cells. In this process, ferumoxytol play a role on macrophage activation and ROS production. It provides a new strategy to induce cell death for more effective cancer treatments. Moreover, leukaemia cells with low FPN expression have little ability to export iron and are susceptible to iron-based oxidative stress, which results in leukaemia cell damage after ferumoxytol administration. This suggests that ferumoxytol can be considered as a feasible strategy for anti-leukaemia therapy. Additionally, the catalytic property of ferumoxytol has been demonstrated, which can generate ROS from hydrogen peroxide via peroxidase-like activity under acidic pH found in pathological conditions associated with dental caries (tooth-decay). It can be used as a nanozyme for pathogen killing and biofilm disruption via selective bacterial binding-activation mechanism to precisely target virulent biofilms associated with severe caries as determined in rodents and in humans. Hence, ferumoxytol-mediated catalytic activation of hydrogen peroxide may be a promising strategy for treating oral infectious diseases. Lastly, the surface coating of ferumoxytol can interact with small molecules through weak electrostatic interactions and accommodate drugs with varying molecular weights. Importantly, MRI can be performed simultaneously to monitor the distribution of ferumoxytol-drug conjugates, while assessing the therapeutic outcome. Thus, ferumoxytol is a theranostic nanoparticle that is both active for MRI and also provides therapeutic effects.

Overall, ferumoxytol has been extensively utilized in biomedical applications both as diagnostics and therapeutics in the last few decades and we expect to see additional advancements of this technology in the near future, particularly as a promising theranostic approach. The research community have been highly inventive in finding ways to use ferumoxytol that go far beyond its originally intended uses. The FDA-approved status can facilitate clinical evaluation of new therapeutic uses in patients. In particular, we expect to see further exploration of ferumoxytol as nanocatalysts for both therapeutics and diagnostics as well as its combinations with other drugs. It may be the case that other FDA-approved iron oxide nanoparticles, such as ferumoxsil, could be similarly repurposed for additional applications, which would be a benefit to the field. Alternatively, other types of FDA-approved nanoparticles, such as Abraxane or Doxil, could see more extensive modification and repurposing. However, further work on the new applications of ferumoxytol and its derivatives needs to be conducted, including additional efficacy and mechanistic studies in humans. In addition, the safety profile of new uses of ferumoxytol need to be investigated in detail. The alternative biomedical applications of ferumoxytol including immunotherapy, anti-leukaemia, anti-biofilm, drug delivery among others are mostly at proof-of-concept stage although robust *in vivo* and some clinical data have been generated. Thus, more research on its therapeutic benefits, potential toxicity, and mechanisms of action needs to be done. We expect that a rich vein of research on this topic will continue to flourish for many years to come, which may lead to additional clinical applications of this versatile multifunctional nanoparticle.

## Figures and Tables

**Table 1:** Examples of iron oxide nanoparticles that have been clinically approved or are currently in clinical trials*.*

Generic Name	Brand Name	Coating	Size	Applications
Ferumoxytol	Feraheme® (US) Rienso® (EU)	Carboxymethy-dextran	17-31 nm	Iron deficiency treatment, MRI contrast
Fermoxtran-10	Combidex® (US) Sinerem® (EU)	Dextran	15-30 nm	MRI contrast
Ferristene	Abdoscan®	Polystyrene	300 nm	MRI contrast
Ferumoxide	Feridex® (US) Endorem® (EU)	Dextran	50-100 nm	MRI contrast
N.A.	NanoTherm®	Aminosilane	12 nm	Cancer treatment
Ferucarbotran	Resovist® (US, EU) Ciavist^TM^ (France)	Carboxydextran	80 nm	MRI contrast
Feruglose	Clariscan^TM^	PEGylated starch	20 nm	Blood pool agent
Ferumoxsil	Lumirem® (US) GastroMARK^TM^ (EU)	Siloxane	300 nm	Oral gastrointestinal imaging

N.A.: Not Available

**Table 2:** Examples of different intravenous iron formulations

Formulation	Brand Name	Carbohydrate	Manufacturer		Concentration of iron (mg/ml)
Ferumoxytol	Feraheme® (US) Rienso® (EU)	Polyglucose sorbitol carboxymethylether	AMAG Pharmaceuticlas	30	
Ferric isomaltoside	Monoferric® (US) Monofer® (EU)	Isomaltoside	Pharmacosmos A/S	100	
Ferric carboxymaltose	Injectafer® (US) Ferinject® (EU)	Carboxymaltose	American Regent	50	
Low molecular weight iron dextran	INFeD® (US) Cosmofer® (EU)	Low molecular weight iron dextran	Watson Pharmaceuticals	50	
Sodium ferric gluconate	Ferrlecit®	Gluconate	Sanofi-Aventis	12.5	
Iron sucrose	Fermed® Venofer®	Sucrose	American Regent	20	
					

**Table 3:** Examples of ferumoxytol in diagnostic, therapeutic, and theranostic applications.

Type of applications	Applications	Indications	Route	Ref.
Diagnosis	HPF was used to label neural stem cells for MRI	Glioma	i.v. injection	71, 73
	Segregation and extravascular localization of ferumoxytol imaging for differentiating extravascular-from-intravascular MRI	Globlastoma	i.v. injection	85
	Ferumoxytol for MR lymphography	Prostate Cancer	i.v. injection	94, 95
	Ferumoxytol-enhanced MRI for vascular applications	Evaluating vascular pathology, presurgical planning	i.v. injection	101
	Multi-modal imaging integrating MRI and PET	Pediatric Cancer	i.v. injection	112, 113
Therapy	Iron supplement for anemic patients	Chronic kidney disease	i.v. injection	19, 20
	Ferumoxytol induced selective cell death in cells that express low levels of ferroportin	Leukaemia	i.p. injection	149
	Ferumoxytol changes the polarization of tumor-associated macrophages to a pro-inflammatory M1 phenotype	MMTV-PyMT cancer, melanoma	i.v. injection	140, 143
	Bacterial pathogen killing and biofilm disruption	Dental caries	Topical oral delivery	13
Theranostics	Ferumoxytol acted as nanovehicle to load drug and monitor drug release using MRI	Prostate cancer, breast cancer	i.v. injection	29
